# Genetic dissection of natural variation in oilseed traits of camelina by whole‐genome resequencing and QTL mapping

**DOI:** 10.1002/tpg2.20110

**Published:** 2021-06-09

**Authors:** Huang Li, Xiao Hu, John T. Lovell, Paul P. Grabowski, Sujan Mamidi, Cindy Chen, Mojgan Amirebrahimi, Indika Kahanda, Brendan Mumey, Kerrie Barry, David Kudrna, Jeremy Schmutz, Jennifer Lachowiec, Chaofu Lu

**Affiliations:** ^1^ Department of Plant Sciences and Plant Pathology Montana State University Bozeman MT 59717 USA; ^2^ School of Computing Montana State University Bozeman MT 59717 USA; ^3^ Genome Sequencing Center HudsonAlpha Institute for Biotechnology Huntsville AL 38508 USA; ^4^ United States Department of Energy Joint Genome Institute Lawrence Berkeley National Laboratory Berkeley CA 94720 USA; ^5^ Arizona Genomics Institute, School of Plant Sciences University of Arizona Tucson AZ 85721 USA

## Abstract

Camelina [*Camelina sativa* (L.) Crantz] is an oilseed crop in the Brassicaceae family that is currently being developed as a source of bioenergy and healthy fatty acids. To facilitate modern breeding efforts through marker‐assisted selection and biotechnology, we evaluated genetic variation among a worldwide collection of 222 camelina accessions. We performed whole‐genome resequencing to obtain single nucleotide polymorphism (SNP) markers and to analyze genomic diversity. We also conducted phenotypic field evaluations in two consecutive seasons for variations in key agronomic traits related to oilseed production such as seed size, oil content (OC), fatty acid composition, and flowering time. We determined the population structure of the camelina accessions using 161,301 SNPs. Further, we identified quantitative trait loci (QTL) and candidate genes controlling the above field‐evaluated traits by genome‐wide association studies (GWAS) complemented with linkage mapping using a recombinant inbred line (RIL) population. Characterization of the natural variation at the genome and phenotypic levels provides valuable resources to camelina genetic studies and crop improvement. The QTL and candidate genes should assist in breeding of advanced camelina varieties that can be integrated into the cropping systems for the production of high yield of oils of desired fatty acid composition.

## Core Ideas

- Genome resequencing and population structure analyses are used to assess genetic diversity.
- QTL mapping is conducted to identify regions associated with seed traits in camelina.
- Genomic resources and candidate genes are provided to improve camelina seed traits.

**Abbreviations**
DTF: days to flowering
DTM: days to maturity
FarmCPU: fixed and random model circulating probability unification
GWAS: genome‐wide association studies
LD: linkage disequilibrium
LOD: logarithm of odds
OC: oil content
PA: pod area
PCA: principal component analyses
PH: plant height
PL: pod length
PW: pod width
QTL: quantitative trait loci
RIL: recombinant inbred line
SA: seed area
SL: seed length
SNP: single nucleotide polymorphism
SPP: seed number per pod
SW: seed width
TSW: 1000‐seed weight.

## 1 INTRODUCTION

Camelina [*Camelina sativa* (L.) Crantz] is a member of the Brassicaceae (or mustard) family (Al‐Shehbaz et al., 2006). It has recently received considerable interest in both oilseed production and plant biology research (Berti et al., 2016). Camelina seed accumulates an oil content (OC) of ∼35% of dry mass, which may provide an important source of bioenergy and other industrial materials (Shonnard et al., 2010). Camelina oils are high (∼50%) in polyunsaturated fatty acids, especially α‐linolenic (18:3), an omega‐3 fatty acid, and are thus also attracting attention for use in the production of health‐promoting foods and feeds (Pilgeram et al., 2007). Agronomic evaluations have indicated that camelina is well‐adapted to the semi‐arid region of the Northern Great Plains and Pacific Northwest (Chen et al., 2015; Obour et al., 2018; Schillinger et al., 2012). By growing as a rotation crop to replace summer fallow in the wheat (*Triticum aestivum* L.)–fallow production systems in this region, camelina would not only provide the feedstock for biofuels, such as a hydroprocessed renewable jet fuel and diesel (Moore et al., 2017; Shonnard et al., 2010), but also potentially improve cereal‐based cropping systems in the U.S. Northwest and boost rural economies (Chen et al., 2015; Obour et al., 2018; Schillinger et al., 2012). Besides its potential in oilseed production, in light of a short life cycle and the ease of genetic transformation (Lu & Kang, 2008), camelina is also an ideal model system for translational plant biology research (Berti et al., 2016; Hines & Travis, 2016; Malik et al., 2018). Thanks to its close relationship to *Arabidopsis thaliana (L.) Heynh*. and the availability of its own genome sequence (Kagale et al., 2014), many traits of economic importance can be effectively tested in camelina by rapid biotechnological approaches including manipulation of gene expression levels (An & Suh, 2015; Nguyen et al., 2013; Y. Zhu et al., 2018) and induction of knock‐out mutants using CRISPR/Cas9 (Aznar‐Moreno & Durrett, 2017; Jiang et al., 2017; Morineau et al., 2017; Ozseyhan et al., 2018). Novel characteristics have also been successfully introduced into camelina by metabolic engineering such as the very‐long‐chain omega‐3 and other high‐value oils (Bansal et al., 2018; Kim et al., 2015; Liu et al., 2015; Usher et al., 2017).

Despite its great potential as an alternative and specialty oilseed crop, camelina is currently not competitive with higher‐yielding crops like canola (*Brassica napus* L.) (George et al., 2017), and several agronomic traits need to be improved to make the production of camelina economically viable (Berti et al., 2016). Among those, the most important ones are traits related to seed oil yield and quality, especially seed size and OC including its fatty acid composition (Berti et al., 2016; King et al., 2019). Improving these traits through conventional breeding or modern biotechnology approaches are hampered by the knowledge gaps in the underlying genetic determinants, which are genetically complex and governed by multiple genes or quantitative trait loci (QTL). Recent advancements in genome science, especially the next‐generation sequencing technologies, have dramatically empowered the dissection of quantitative traits (Nguyen et al., 2019). In camelina, limited studies have found wide ranges of genetic and trait variations among different genotypes including natural accessions and breeding lines, which may provide valuable resources to understand the genetic mechanisms underlying its natural variation and to improve the agronomics and productivity of this crop (Hotton et al., 2020; Luo et al., 2019; Vollmann et al., 2007).

**Core Ideas**

- Genome resequencing and population structure analyses are used to assess genetic diversity.
- QTL mapping is conducted to identify regions associated with seed traits in camelina.
- Genomic resources and candidate genes are provided to improve camelina seed traits.

The major goals of this research are to (a) to evaluate the genetic variation in a diverse panel of camelina accessions at the genotypic and phenotypic levels and (b) dissect some of the most important traits associated with camelina productivity using quantitative genetics. We characterized a worldwide collection of 222 camelina accessions using high‐density single nucleotide polymorphisms (SNPs) generated by whole‐genome resequencing. Population structure analysis revealed that the collection can be clustered into four different groups in agreement with their phylogeny and geographic origins. Further, incorporating phenotypic variation revealed by field evaluation, we performed genome‐wide association studies (GWAS) and linkage mapping on the diverse panel and a recombinant inbred line (RIL) population. We identified several QTL and major candidate genes associated with key determinants of seed yield including seed size and OC, as well as those for fatty acid composition and flowering time. This study indicates that the natural germplasm is a valuable resource that can be exploited for camelina research and crop improvement. Our genetic mapping results also provide some of the most impactful alleles to breed advanced camelina varieties that can be integrated in the cropping systems for the production of oils with desired fatty acid profiles.

## 2 MATERIALS AND METHODS

### 2.1 Plant materials and field experiments

The diversity panel used in this study was comprised of 222 camelina accessions, mainly from the collections in the Leibniz Institute of Plant Genetics and Crop Plant Research and the U.S National Plant Germplasm System. Detailed information including their names, biological status, and country of origin is listed in Supplemental Table S1. The RIL population consisting of 257 lines was developed by single‐seed descent method from a cross between a large‐seed accession Pryzeth and a cultivar Suneson at Montana State University (King et al., 2019). All accessions and F_5‐6_ RILs evaluated in this study were cultivated in field trials at the Arthur H. Post Research Farm near Bozeman, MT, USA (45°67′ N, 111°15′ W) during the 2017 and 2018 growing seasons. Each accession and RIL was grown as a single replication in two 3‐m rows with a distance of 0.3 m between rows within a randomized complete‐block design.

### 2.2 Measurement of agronomic and seed traits

Days to flowering (DTF) and maturity (DTM) were recorded in the field as the number of days from planting to the date when 50% of the plants in a plot had showed the first flower and when 95% of the pods had ripened as indicated by brown pod color, respectively. Plant height (PH) was the average of three measurements per plot, and each measurement was recorded as the length of main stem from the ground to the top of the plant at maturity. Mature pods and seeds were harvested from five randomly selected plants in the middle of each plot for postharvesting measurements including pod size, seed size, and seed weight. Seeds and pods of each accession and RIL progeny were imaged on an office scanner (Epson Perfection V600) with resolution setting at 600 dpi. Image files were processed on SmartGrain (Tanabata et al., 2012) to obtain morphological perimeters (length, width, area) as raw phenotypic data. Seed OC was determined by a benchtop nuclear magnetic resonance seed analyzer (MQC23, Oxford Instruments) and presented as percentages of seed mass. Relative abundance and composition of seed fatty acids was determined by gas chromatography on a Shimadzu 2010 GC system with an Agilent HP‐Innowax column (Snapp et al., 2014). Best linear unbiased prediction values of each trait from all accessions and RILs in 2017 and 2018 field trials were calculated using the R package lme4 (Bates et al., 2015) and used for association mapping and QTL analysis, respectively. Broad‐sense heritability (*H* ^2^) of each trait and Pearson's correlation coefficients between different traits were computed using the SOMMER package (Covarrubias‐Pazaran, 2016) and the cor() function in R.

### 2.3 Genome resequencing and SNP calling

For genome resequencing, high‐quality DNA was extracted from young leaves from a single plant of each accession and RIL using the high‐throughput DNA extraction platform at the Arizona Genomics Institute at the University of Arizona. Whole‐genome resequencing was performed on Illumina Novaseq S4 for paired‐end reads of 2 by 150 kb with an average genome coverage of 35.5× and 13.4× for accessions and RILs, respectively. Sequencing reads were screened for PhiX, mitochondria, and chloroplast contamination. Reads were removed if they contained >95% simple sequence content or were <50 bp after trimming adapters and low‐quality (*q* < 20) bases. The reads were then aligned to the *Camelina sativa* v2 reference genome (Kagale et al., 2014) using Burrows–Wheeler alignment tool (Li & Durbin, 2009) and the ‐M flag default mapping parameters. A pileup file was generated using the alignments of all the samples and the SAMtools pileup function (Li et al., 2009) during which, secondary alignments were excluded. Multiple primary alignments (same highest score) were tolerated with the understanding that regions of low divergence between homeologs may contain some wrongly mapped variation, reducing the power to make linkage‐based inferences in these regions. The pileup file was used with VarScan (Koboldt et al., 2009) to discover SNPs and call genotypes across all samples. The SNPs (>4 million) were filtered in VCFtools (Danecek et al., 2011) to retain biallelic SNPs with missing data <10%, and heterozygosity <0.5. SNPs were then pruned using PLINK (Chang et al., 2015), retaining SNPs (∼166,000) with linkage disequilibrium (LD) *r* ^2^ < 0.4 and minor allele frequencies (MAF) >0.1. In addition, average pairwise differences (π) and fixation index (*Fst*) were calculated in VCFtools to estimate the genetic diversity based on the genome‐wide SNPs information (MAF >0.01). Sequences of the camelina accessions have been deposited into the NCBI database (accessions SRP215681‐220853; 241487–241525).

For the RIL population, the resulting genotype matrix was filtered from 514,343 markers to the 434,945 markers with <25% missing data. Six libraries (individual RILs) with highly distorted markers with MAF averaging <0.2 were dropped. We then subset markers to those with MAF >0.25, resulting in a culled input matrix of 402,838 markers and 257 libraries. To produce an accurate genetic map, we need to retain nonredundant markers that minimize the amount of missing data. To do this, we employed a two‐step approach in the R package SNPRelate. First, we calculated the maximum observed LD using a 50‐marker sliding window for each marker with its 49 physically proximate markers. If a marker is never in perfect LD with its neighbors, this is indicative of genotyping errors; 394,504 markers passed this step. Second, we pruned the matrix to 28,521 nonredundant markers (LD ≤ 0.999). R/qtl (Broman et al., 2003) and R/qtlTools (https://github.com/jtlovell/qtlTools) were further used to prune the genetic map for each chromosome and scaffold based on the physical order of markers. After dropping noninformative markers, the resulting map contained 3,070 markers for linkage analysis. We formed 20 linkage groups using a maximum recombination fraction of 0.23 and a minimum logarithm of odds (LOD) score of 3. Markers were ordered within linkage groups using the Concorde traveling salesperson solver via TSPmap (Monroe et al., 2017). After culling and fine tuning the marker order through the crossover‐counting ripple algorithm with a window of five markers, we visually inspected and dropped seven markers that dramatically increased the map length. A final pruning was conducted as above, resulting in a nearly saturated 2,593‐marker map. Sequences of the RILs have been deposited into the NCBI database (accessions SRP236608‐236871).

### 2.4 Phylogeny, population structure, and LD

The final SNP dataset of accessions with 161,301 SNPs was used to construct the maximum likelihood tree using IQ‐TREE v1.6.6 with the best model GTR+F+ASC+R5 determined by the Bayesian information criterion. The consensus phylogenetic tree was visualized in MEGA4 (Tamura et al., 2007). Population structure analysis was conducted with fastSTRUCTURE (Raj et al., 2014) and ADMIXTURE (Alexander et al., 2009) software on the final dataset at *K* values from 1 to 32 to find the optimal *K*, which indicates the most likely number of clusters in the accessions. We used the filtered SNPs to estimate LD across camelina genome with the software package TASSEL 5 (Bradbury et al., 2007). The squared correlation coefficient (*r* ^2^) of alleles was calculated and plotted with R scripts, which drew average *r* ^2^ (*y* axis) against the means of the distances between SNP pairs along the *x* axis. The LD decay rate of the diversity panel was measured as the physical distance (kb) where the average *r* ^2^ value dropped to half its maximum.

### 2.5 GWAS and QTL analysis

Association mapping on camelina accessions was conducted using the generalized linear model, the mixed linear model, the compressed mixed linear model, and the fixed and random model circulating probability unification (FarmCPU) model, implemented with the GAPIT R package (Lipka et al., 2012; Tang et al., 2016). Quantile–quantile plots were generated to evaluate the fit of each model for the traits. For the FarmCPU analysis, the first four axes of the principal component analyses (PCA) estimated in TASSEL were used as covariates. The Bonferroni correction was used to determine the significance of marker–trait associations based on a nominal level of α = 0.05, corresponding to a −log_10_ (*P*) value of 6.51 in this study.

For linkage mapping, we performed a series of QTL analyses including interval mapping, cofactor selection, and permutation testing using QGene 4.4.0v (Joehanes & Nelson, 2008) as previously described (King et al., 2019). Candidate genes for the trait of interest were predicted using the QTL interval locations or the estimated LD regions with strongest associated SNPs in the GWAS. The genomic locations were queried against the camelina reference genome (Kagale et al., 2014) to identify genes that have known functions associated with the trait.

## 3 RESULTS

### 3.1 Population structure and genomic variation of a camelina germplasm panel

**FIGURE 1:**
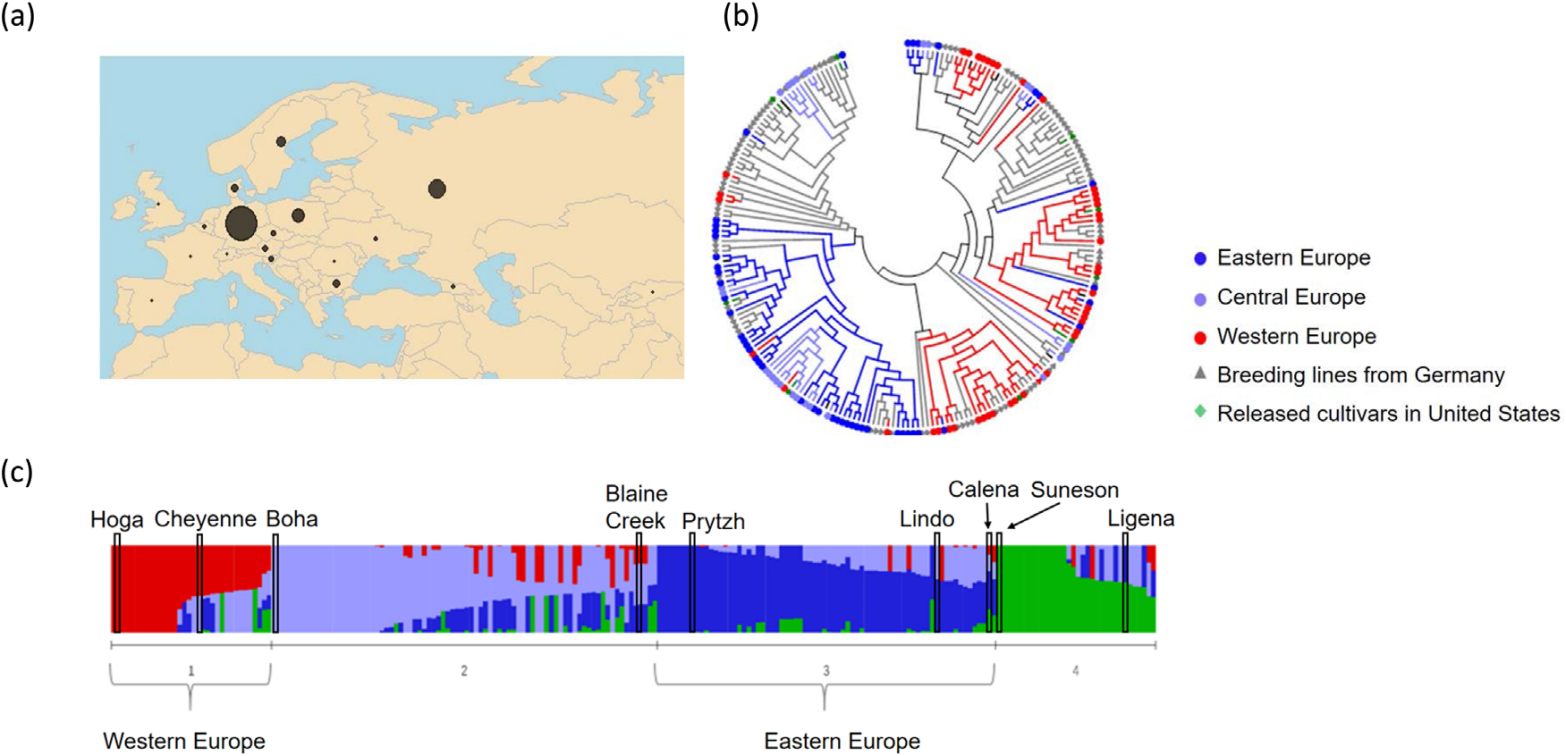
Geographic distribution, phylogeny, and population structure of *Camelina sativa* accessions. (a) Geographic distribution of resequenced camelina accessions in Europe and western Asia. The diameter of the black circle is proportional to the number of accessions. (b) Maximum likelihood tree of all accessions inferred from single nucleotide polymorphisms. (c) Model‐based Bayesian clustering of all accessions performed using fastSTRUCTURE with the optimal number of ancestry kinship (*K*) set to 4. Names of some camelina cultivars are labelled on top

A population of 213 camelina accessions was collected and evaluated previously (Luo et al., 2019). The accessions represented geographical distributions across 19 different countries in Europe and Asia (Figure 1a). We obtained this population and added several breeding lines and released cultivars from North America for a total of 254 accessions. We conducted genotyping of all accessions by whole‐genome resequencing at an average depth of 35.5×, eliminated putative duplicates, and used 222 nonredundant accessions for subsequent studies. After data processing and filtering, we obtained 161,301 high‐quality biallelic polymorphism markers (see Methods for criteria) based on the camelina reference genome (Kagale et al., 2014). The average density across the genome was one marker per 3.8 kb. Information on the number of filtered markers, map length, and filtered marker density for each chromosome are presented in Supplemental Figure S1.

We performed population structure analysis using these markers with ADMIXTURE (Alexander et al., 2015) and fastSTRUCTURE (Raj et al., 2014) to examine the relationships among accessions. Based on the consensus results of minimal cross‐validation error of different *K*‐value settings (Supplemental Figure S2), we found that the accessions clustered into four groups (*k* = 4), roughly reflecting the geographical distribution of camelina genotypes. The results from the population structure analysis were mirrored in the generation of a maximum likelihood tree displaying the phylogenetic relationships among the 222 camelina genotypes. Most accessions from eastern European countries were clustered into groups distantly related to groups of western European accessions (Figure 1b). Using the same set of SNP markers, the average LD decay distance of the whole genome in all accessions was estimated at 593 kb, where *r* ^2^ = 0.24 (half of its maximum value) (Supplemental Figure S3).

While a considerable number of breeding lines developed in Germany are present in all four subpopulations, we observed a western–central–eastern European continuum pattern of accessions corresponding to their country of origins (Figure 1c). Western European cultivars, including some from Sweden and Denmark, two countries with the earliest record of camelina breeding efforts, congregate in subpopulation 1 and 2. Most cultivars from Russian and eastern Europe belong to subpopulation 3, while accessions collected from the central part of the European continent cluster into subpopulation 3 and 4. In general, the individuals from the adjacent geographical regions were clustered more tightly with each other than with individuals from distant countries. Notably, breeding lines from Germany and released cultivars of North America present in different groups also exhibit high levels of population group admixture. For example, the well‐adapted cultivar Calena, selected for high OC and field performance in North America, was admixed among subpopulations 1, 2, 3, and 4, with a higher contribution from subpopulation 3. Other lines, including cultivars from Germany (‘Lindo’ and ‘Ligena’), the early bred lines adapted to northern Europe (‘Boha’ and ‘Hoga’), and released cultivars in the United States (‘Cheyenne’ and ‘Blaine Creek’) are present in different subpopulations, reflecting their diverse backgrounds.

### 3.2 Evaluation of phenotypic variation and correlations of camelina oilseed traits

**FIGURE 2:**
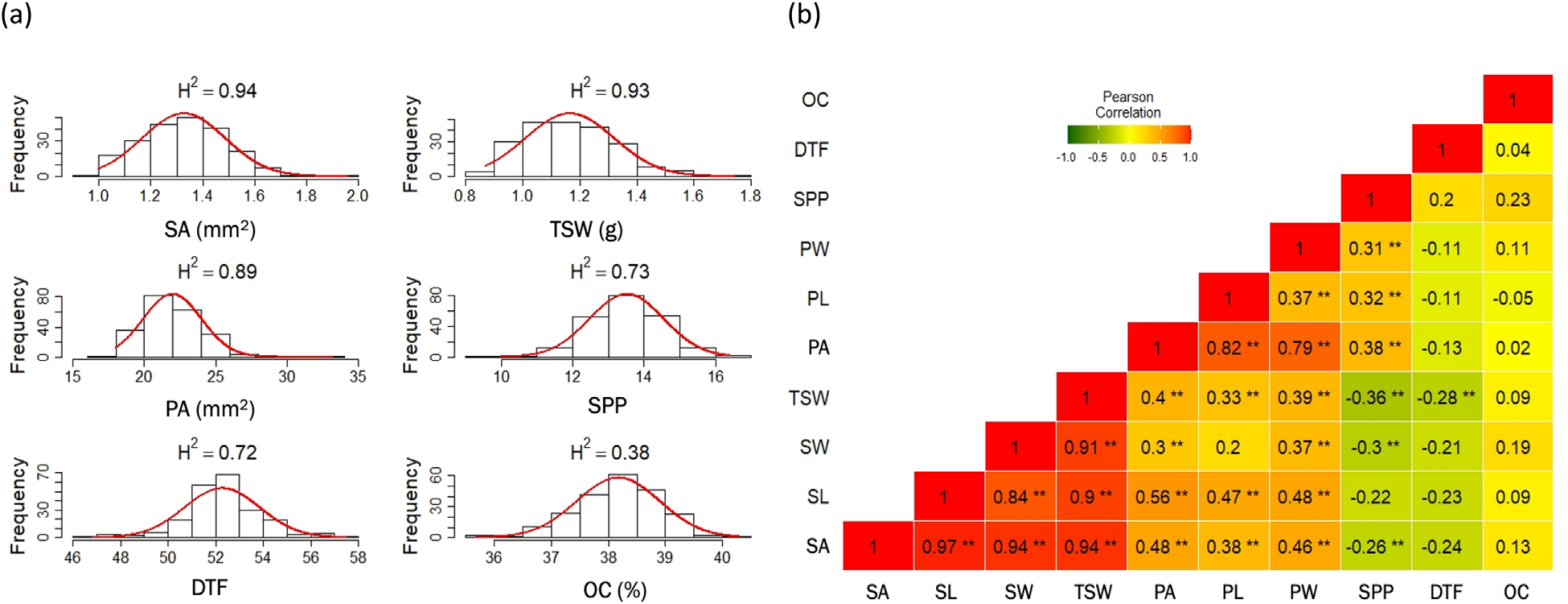
Phenotypic variation and correlation of key oilseed traits in camelina accessions. (a) Histograms of the distribution of seed area (SA), 1000‐seed weight (TSW), pod area (PA), seed number per pod (SPP), days to flowering (DTF), and seed oil content (OC). Broad‐sense heritability (*H* ^2^) of each trait is shown on the top of its histogram. (b) Heatmap of the Pearson correlation of these traits, along with the size parameters: seed length (SL), seed width (SW), pod length (PL), and pod width (PW)

We grew all genotyped 222 camelina accessions in the field in 2017 and 2018 using a randomized complete‐block design (King et al., 2019) to evaluate key traits related to seed production. Near‐normal distribution and highly heritable (*H* ^2^ >0.7; Figure 2a) phenotypic variation in seed area (SA) and pod area (PA), 1000‐seed weight (TSW), seed number per pod (SPP), and DTF was observed among these accessions. Compared with DTF and size‐related traits, PH and seed OC (OC) had relatively lower heritability (*H* ^2^ = 0.22 and 0.38, respectively). All traits exhibited moderate‐to‐high correlations between two growing seasons ranging from 0.35 to 0.86 (all *P* < .001). Descriptive statistics of all measured traits, including the mean, range, year‐to‐year correlation, and heritability is shown in Supplemental Table S2.

Pairwise phenotypic correlations between different traits were calculated as the Pearson's correlation coefficients of their best linear unbiased prediction values (Figure 2b). First, we observed high correlations among SA, seed length (SL), and seed width (SW), suggesting a potentially high degree of shared genetic basis in these three size parameters. Similarly, PA was highly correlated to pod length (PL) and pod width (PW). As we previously reported in a biparental population (King et al., 2019), SA was also correlated with TSW (*r* = 0.94) and PA (*r* = 0.48) in the accessions. Seed number per pod, as a trait likely influenced by the interaction between seed size and the capacity of pod, showed a negative correlation with SA (*r* = −0.26) and a positive correlation with PA (*r* = 0.38). Days to flowering showed a negative correlation with TSW at a modest level (*r* = −0.28) but weak correlations with other seed and pod size parameters. No significant correlation was found between OC and seed size‐related traits in the accessions of this study.

### 3.3 Association mapping of key oilseed traits

To dissect the genetic basis of natural variation in camelina oilseed traits, we performed GWAS within the diversity panel. Based on the evaluation of different statistical methods by quantile–quantile plots, we found FarmCPU exhibited higher statistical power than the mixed linear model and compressed mixed linear model approaches, especially for the association analyses of seed size and OC in this study (Supplemental Figure S4). FarmCPU has been proposed and empirically verified in several other studies (Tan & Ingvarsson, 2019; Tao et al., 2020; X. M. Zhu et al., 2018) for dissecting complex traits by efficiently addressing the confounding problems between testing markers and covariates and controlling false positives. It is worth noting that Manhattan plots generated by FarmCPU only display the most significant SNP among a cluster of SNPs within proximity, rather than columns of SNPs resembling the Manhattan skylines showed by the traditional linear models (Liu et al., 2016).

#### 3.3.1 QTL for seed size in the diversity panel

**FIGURE 3:**
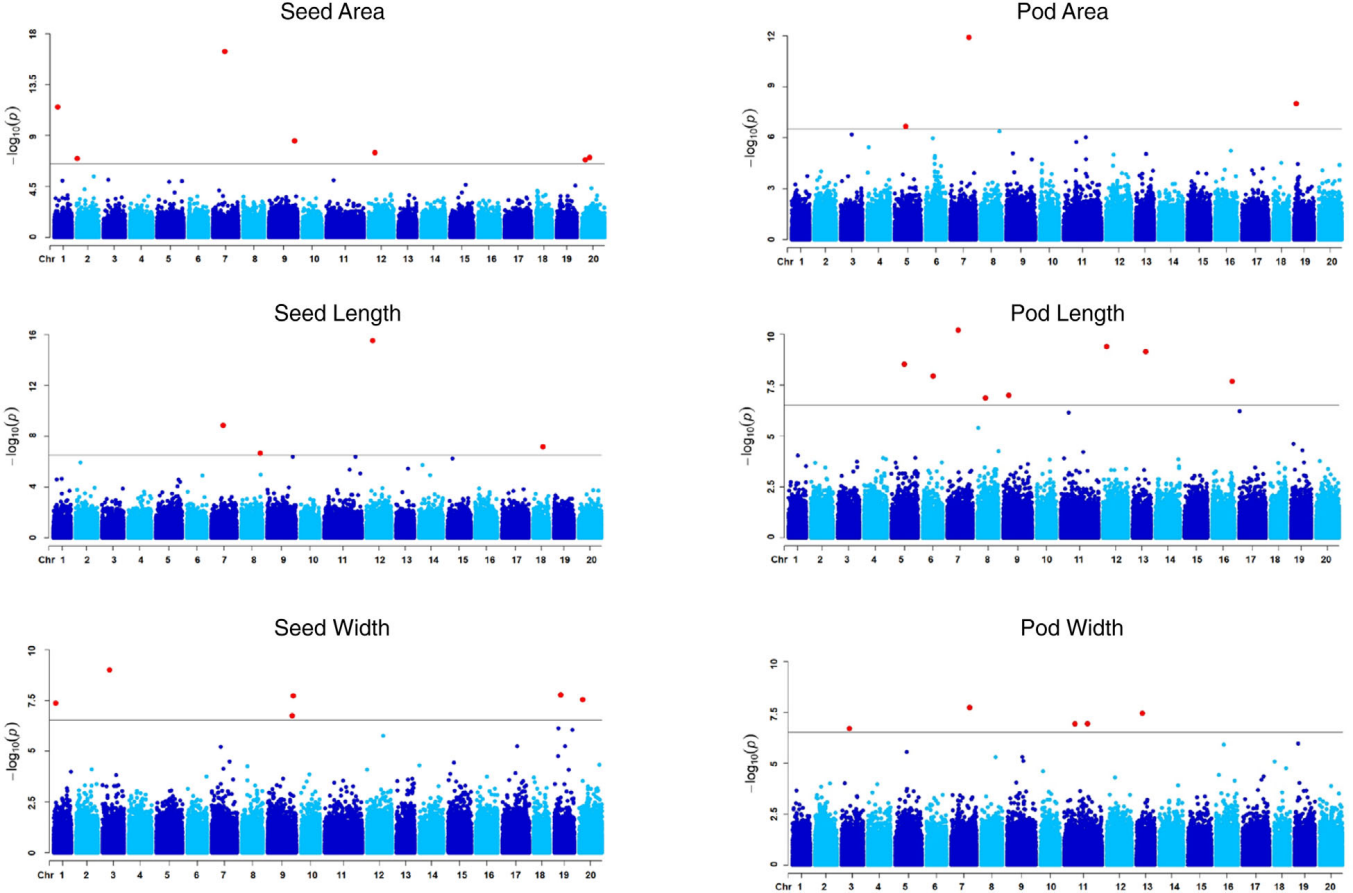
Genome‐wide association study (GWAS) of seed and pod size parameters in the diversity panel. The GWAS results are visualized in Manhattan plots. Associations reaching genome‐wide significance threshold of −log(P) ≥6.51 (horizontal black line) are shown in red. For each cluster of significant single nucleotide polymorphisms, only the one with the highest association is displayed in these plots using the fixed and random model circulating probability unification (FarmCPU) model

As a critical yield‐determining component, seed size was measured as three size parameters (area, length, and width) together with related traits including pod size parameters, TSW and SPP. Using the FarmCPU approach, we identified a total of 47 significant QTL associated with seed‐size‐related traits (Figure 3; Supplemental Table S3). Among them, eight QTL were found to be collocated and shared two or more traits. Phenotypically correlated parameters of seed and pod size shared a number of identified SNP associations. For example, SA and SL shared significantly associated SNPs on chromosome 7 and 12. On the other hand, dissection of the overall seed and pod sizes to simpler parameters (length and width) identified new associations, which may have fallen below the significance threshold and then been overlooked. For example, SNP peaks on chromosome 13 were identified with significant associations with PL and PW while absent in PAs. In addition, we detected seven significant QTL for TSW with the highest associated SNP on chromosome 20. For SPP, seven QTL were identified on six chromosomes. The information of all GWAS‐detected QTL including the position of lead SNP, *P* value, MAF, and SNP effect is listed in Supplemental Table S3.

#### 3.3.2 QTL for fatty acid composition and seed oil content

**FIGURE 4:**
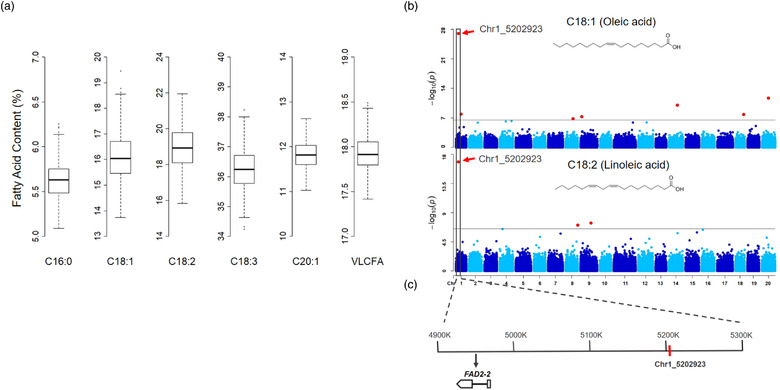
Genome‐wide association study (GWAS) on fatty acid composition of camelina accessions locates the region harboring the *FAD2‐2* gene. (a) Box plots of seed fatty acid content variation in the camelina accessions. (b) Manhattan plots for GWAS of the oleic acid and linoleic acid contents. The overlapping GWAS signals detected for both fatty acid components are highlighted by a column, and the strongest associated SNP (Chr1_5202923) is marked by red arrows. (c) The camelina *FAD2‐2* gene (*Csa01g013220*; Chr1:4949323‐4952363) marked by an arrow is located around 250 kb of the highly associated single nucleotide polymorphism (red bar)

Many camelina breeding efforts have focused on increasing OC and improving the fatty acid profile for different dietary and industrial applications. To explore potential gains of high‐value genetic loci for seed oil, we performed GWAS for the total seed OC and the concentration of five major fatty acids that are present in camelina seeds: palmitic (C16:0 = number of fatty acyl C‐chain length:double bonds), oleic (C18:1), linoleic (C18:2), linolenic (C18:3), and eicosenoic (C20:1) acid in all accessions. Traits were measured by nuclear magnetic resonance and gas chromatograph described in our previous reports (Kang et al., 2011; King et al., 2019). Four unsaturated fatty acids (C18:1, C18:2, C18:3, and C20:1) comprised an average of 83.2% of the total seed oil, and of the saturated fatty acids, C16:0 had the highest mean proportion (5.6%). Unsaturated fatty acid C20:1 was the most abundant component of the very‐long‐chain fatty acids with an average of 11.8% of the total OC. In comparison, three major C18 fatty acids (C18:1, C18:2, C18:3) exhibited the largest variation among the accessions (Figure 4a), and they were negatively correlated with each other (Supplemental Figure S5). Oil content showed positive correlations with the content of C18:1 but a negative correlation with C18:3 (Supplemental Figure S5). By GWAS, we detected a very strong association signal on chromosome 1 for the oleic acid (C18:1) with a −log_10_ *P* value of 26.98 (Figure 4b). Further analysis identified that the lead SNP (Chr1_5202923) was located within the estimated LD region (∼253 kb) that harbors the *FAD2‐2* gene (*Csa01g013220*) (Figure 4c). *Csa01g013220* is an ortholog of *A. thaliana FAD2* (*At3g12120*), which encodes an endoplasmic reticulum localized omega‐6 desaturase responsible for the desaturation of C18:1 to C18:2, the critical enzymatic step of polyunsaturated lipid synthesis in seeds. Expectedly, this SNP was also highly associated with the linoleic acid (C18:2) (−log_10_ *P* = 16.84) with an opposite effect (Figure 4b; Supplemental Table S3), supporting that *FAD2‐2* may be the major causative gene underlying the variation of these two fatty acid components in camelina accessions.

**FIGURE 5:**
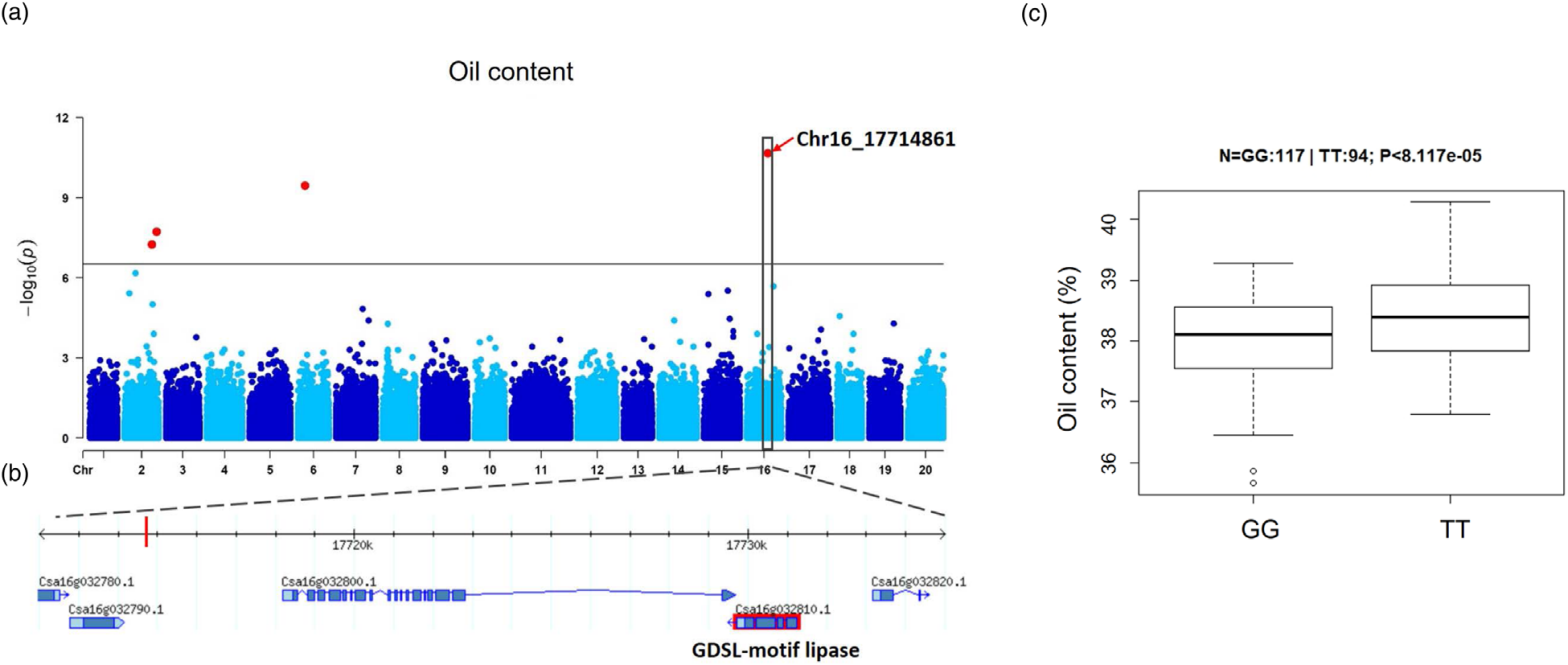
A GDSL gene is a candidate for oil content variation in camelina accessions. (a) Manhattan plot for genome‐wide association study of oil content. The detected region on chromosome 16 is highlighted by a column and the strongest associated single nucleotide polymorphism (SNP) (Chr16_17714861) is marked by a red arrow. (b) Zoomed‐in view of the strongest associated SNP on the physical map of chromosome 16. Its position is marked by a red bar. The candidate gene GDSL‐motif lipase (*Csa16g032810.1*) is highlighted in red. (c) Box plot of oil content comparison between haplotypes in the accessions. The center line indicates the median; box limits indicates the range of the 25th to 75th percentiles of the total data; whiskers indicate the interquartile range; points show outliers

Similarly, we performed GWAS on the seed OC and identified four associated SNPs on three different chromosomes above the significance threshold of −log_10_ *P =* 6.51. Of these, the most significant SNP was Chr16_17714861 (−log_10_ *P* = 10.67) (Figure 5a; Supplemental Table S3). Further analysis found this SNP was located close to the gene encoding a GDSL‐motif lipase (*Csa16g032810*) (Figure 5b). In addition, a strong association of this candidate gene to seed OC was suggested by the haplotype analysis of the above SNP (Chr16_17714861). Haplotype with the allele GG was mainly found in low‐oil‐content accessions, whereas haplotype with TT allele was found in high‐oil accessions (Figure 5c). Other QTL and candidate loci associated with OC and fatty acids are listed in Supplemental Table S3.

### 3.4 Linkage mapping of seed quality and field traits

The above results clearly indicated that putative QTL associated with camelina seed traits can be identified by the GWAS approach. However, it is also evident that GWAS has limited power to detect rare alleles that occur at a low frequency in a natural population. For example, we were unable to detect any significant loci for the variation of flowering time in the diversity panel. Previously, we have developed a RIL population by crossing two contrasting lines, Suneson and Prytzh, which differ significantly in several important traits including SA/PA and OC, as well as DTF, DTM, and PH. Multiple QTL of SA and OC were mapped using the genotyping‐by‐sequencing data on a subset (189 RILs) of the population (King et al., 2019). To complement the association mapping results from the present study and gain a comprehensive understanding of key camelina field and seed traits, here we improved the linkage mapping with a high‐density map and conducted additional trait evaluations of an expanded RIL population. Specifically, we performed whole‐genome resequencing of 257 RILs at an average of ∼13.4× depth and constructed a near‐saturation linkage map with 2,593 high‐quality SNPs. Using measurements of 12 field and seed quality traits from 2‐yr field trials, we identified QTL in the RIL population for these two categories.

#### 3.4.1 QTL for seed size and quality traits

In the RIL population, seed quality traits (SA, PA, TSW, SPP, and OC) and size parameters (SL, SW, PL, and PW) displayed a wide range of distribution with moderate to high heritability (*H* ^2^ = 0.64∼0.89) in field trials (Supplemental Table S4). Transgressive segregation was observed in the progeny, where some RILs have exceeded in SPP and OC as compared with the parental lines. However, no transgressive segregation was observed for SA, PA, and TSW. Compared with the phenotypic correlation analyses in the diversity panel, similar correlation patterns were observed among seed and pod size parameters, TSW and SPP in the RILs. Interestingly, OC was found to be negatively correlated with SA (*r* = −0.32) and positively correlated with SPP (*r* = 0.37) in this biparental population (Supplemental Figure S6).

**FIGURE 6:**
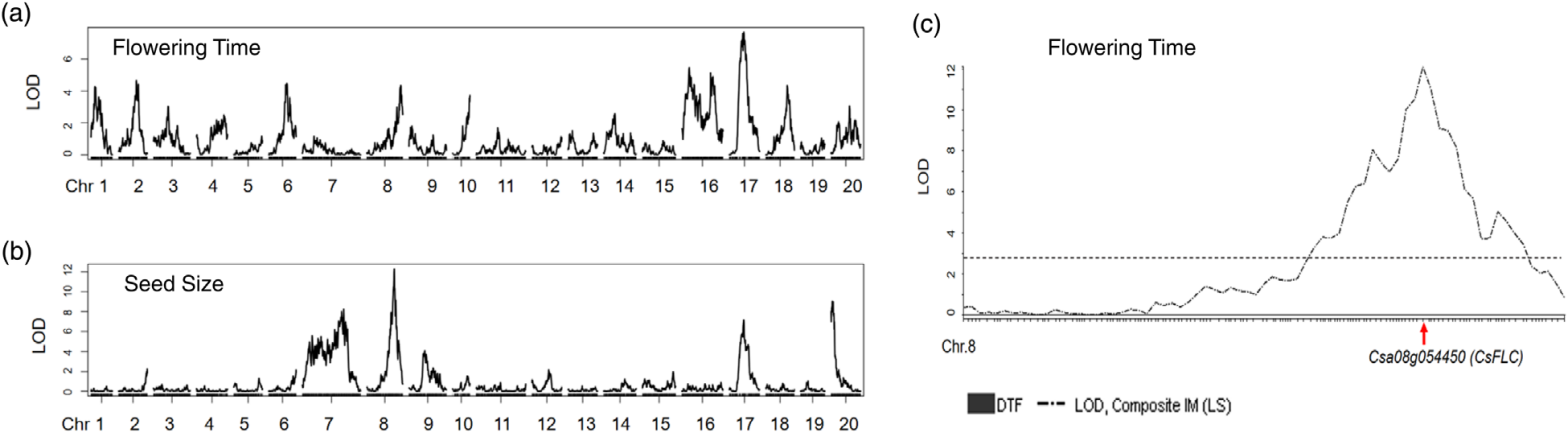
Quantitative trait loci (QTL) analysis in the recombinant inbred line population for seed size and flowering time. (a) Genomic scan of QTL of seed size. (b) Genomic scan of QTL of flowering time. (c) Zoomed‐in view of the QTL profile on chromosome 8 for flowering time. The location of Cs*FLC* (*Csa08g054450*; Chr8:23298987..23304974), a major gene for flowering time is marked by a red arrow. The dotted horizontal line indicates the threshold determined by permutation tests at a significance level of .01

Multiple QTL of SA, PA, and TSW were identified across the genome by composite interval mapping (Figure 6a; Supplemental Table S5), reflecting their polygenic nature. We also observed a high degree of QTL colocalization for these traits. Among them, a major QTL on chromosome 17 was consistently colocalized for SA, SL, PL, and TSW, in agreement with our previous study (Supplemental Table S5). Delineation of this region with SNPs in this study was able to narrow down this interval from the previous 6 cM to the current 1.5 cM, corresponding to a physical distance of 262 kb. Two significant QTL for seed OC were identified on chromosome 2 and 9, explaining a total of 31.3% of the phenotypic variation in the RIL population (Supplemental Table S5).

#### 3.4.2 QTL for the flowering time and plant height

Flowering time is one of the most critical traits associated with plant fitness. It is particularly important for camelina breeding in order to adapt into existing cropping systems of the specific climate (Anderson et al., 2018). We recorded DTF, DTM, and PH in the field. The parental lines Suneson and Pryzth differed in DTF by 4.7 d, and the RIL population showed a wide variation in DTF, ranging from 39.8 to 52.5 d (Supplemental Table S4). We identified three QTL for DTF and DTM on chromosome 7, 8, and 17 by composite interval mapping (Figure 6b; Supplemental Table S5). Among them, the QTL on chromosome 8 was the most predominant (LOD = 12.4). Further analysis found this QTL spanned a region of 342 kb and its peak corresponded to a camelina *Flowering Locus C* (*FLC*) gene (*Csa08g054450*) (Figure 6c). The QTL on chromosome 17 for DTF and DTM was colocalized with the major QTL for seed‐size‐related traits mentioned above, making it the most pleiotropic region in this study that merits further investigation.

Plant height is a major factor of crop plant architecture and plays a critical role in its agronomic performance. In this study, five major QTL for PH were detected and accounted for 6.9–17.7% of the phenotypic variation. Of these, a QTL on chromosome 7 was colocalized with DTF and DTM (Supplemental Table S5).

## 4 DISCUSSION

Camelina is a promising oilseed crop that may provide an important resource of industrial oils and health‐promoting omega‐3 fatty acids. It is generally planted in the spring, although some varieties can be grown as a winter crop (Anderson et al., 2018; Luo et al., 2019). Because of the fallout of favoring more productive oilseed crops, such as canola, the breeding effort in camelina has been, until recently, limited (Berti et al., 2016). Exploitation of natural variation and modern genomics tools may accelerate the breeding approaches to improve the productivity, adaptation, and oil quality in camelina. In this study, we analyzed the genotypic and phenotypic variation in a population of predominately spring‐type camelina.

### 4.1 Genetic potential of improving the camelina spring biotype

Camelina originated in vast regions across southeast Europe and southwest Asia (Brock et al., 2018). Previous studies suggested that domesticated camelina, which includes primarily spring types, has limited genetic diversity and therefore may limit the potential of crop improvement (Chaudhary et al., 2020; Ghamkhar et al., 2010; Manca et al., 2013; Singh et al., 2015; Vollmann et al., 2005). These studies, however, suffered from small numbers of accessions and molecular markers (e.g., random amplified polymorphic DNA, amplified fragment length polymorphism, SSR, and SNP). Here, we collected and assessed 222 camelina accessions by phenotypic evaluation in field trials and genotyping using whole‐genome‐resequencing‐derived, high‐density SNPs.

In field experiments, we observed wide ranges of variation in various plant growth and seed traits (Figure 2). Similar to results we obtained before in a RIL population (King et al., 2019), high correlations were detected among traits such as pod size, seed size, and seed weight. Interestingly, we found that TSW was negatively correlated with DTF (*r* = −0.28, *P* < .01) and with DTM (*r* = −0.16) though not statistically significant despite the high correlation between the DTF and DTM (*r* = 0.55, *P* < .01). Although statistically insignificant and are not presented in Figure 2, we found that the DTF was negatively correlated (*r* = −0.15) with the seed maturation period (from flowering to maturation), and the TSW was positively correlated (*r* = 0.10) with DTM. Therefore, an extended seed maturation period may attribute to the larger TSW. These results suggest that it is possible to breed early flowering varieties of camelina without compromising seed weight. Future experiments are needed to determine the relationship between DTF and TSW and its underlying mechanisms, which may provide new breeding strategies to improve seed yield and agronomics in camelina.

The field trial results suggest that wide phenotypic variations may be associated with genotypic diversity in the natural population. Based on genome‐wide SNP information, we estimated the genetic diversity by calculating the pairwise differences (π), the global fixation index (Fst), and pairwise Fst values among those subpopulations. The π value for all camelina accessions was estimated to 0.0013, which is comparable to π estimated in natural *A. thaliana* lines (Alonso‐Blanco et al., 2016). While the level of diversity (global Fst = 0.112) is lower than other domesticated crops, we did find the Fst values are much higher than previous studies of camelina (Chaudhary et al., 2020; Luo et al., 2019), which might have underestimated the genetic diversity of camelina because of a limited number of molecular markers and accessions. We also observed a clear pattern of genetic divergence in population structure and PCA. Pairwise Fst estimates also revealed that subpopulation 4 has the highest divergence from other groups, in agreement with the PCA result showing a distinct cluster of individuals from group 4 (Supplemental Figure S2).

Based on genotype analyses and trait evaluations in the field, we conclude that our camelina collection has a modest degree of genome diversity, similar to canola (Wu et al., 2019). Nevertheless, the population still provides a wide range of genetic potential for purposes of both breeding and biological studies. Indeed, as discussed below, we were able to identify QTL controlling several traits by GWAS using the accessions. However, our study also suggests that GWAS with a population of modest genetic diversity has limited power in gaining a comprehensive understanding of genetic architectures of those traits in camelina, which may have complex genetic architectures controlled by many loci of small effects. We therefore demonstrated that linkage mapping by crossing contrasting lines that differ in genes of interest may complement the efforts to identify additional QTL and pinpoint candidate causal genes. Previously, we developed a RIL population from two parental lines (Suneson and Pryzth) with striking differences in traits, for example, seed size, and performed a QTL mapping study on a subset of population (189 RILs) with genotyping‐by‐sequencing data (King et al., 2019). In this study, we found that Suneson and Pryzth belong to different subpopulations by population structure analysis. The diversity allowed us to obtain a high‐density linkage map by genome resequencing of a larger RIL population (257 RILs). Consequently, we successfully mapped QTL regions with improved resolution using more SNP markers. For instance, a previously identified major QTL region (∼1.7 Mb) on chromosome 7 for seed‐size‐related traits was delineated to a region of 262 kb (Figure 6a). In addition, expanding the phenotyping by including critical field traits has pinpointed some putative candidate genes such as the *FLC* for flowering time (Figure 6c). Future mapping studies may benefit from a greater genetic diversity in populations derived from crosses among multiple accessions, such as the Multi‐parent Advanced Generation Inter‐cross (MAGIC) (Cavanagh et al., 2008) and the Nested Association Mapping (NAM) (McMullen et al., 2009). To this end, our genotyping through resequencing results (Figure 1) may provide guidelines for future designs of the mapping population construction.

### 4.2 *CsFAD2‐2* is a key determinant of fatty acid composition in camelina seed oils

Fatty acid composition is the determining factor of a vegetable oil for its uses and value; therefore, optimizing the seed oil fatty acid composition is one of the most important objectives for oilseed breeding. Most commodity vegetable oils usually contain fatty acids of 16–18 C atoms and up to three unsaturated double bonds, although variations of C‐chain length and special functional groups are widely present in nature (Ohlrogge et al., 2018). Our evaluation of the camelina accessions indicated that the most significant variation is in the level of unsaturation, that is, the concentration of the omega‐6 (linoleic; C18:2) and the omega‐3 (α‐linolenic; C18:3) polyunsaturated fatty acids (Figure 4a). These fatty acids are synthesized by the sequential actions of the fatty acid desaturase (FAD2), which desaturates the oleic acid (C18:1) into C18:2, with FAD3 performing a further desaturation into C18:3, in the endoplasmic reticulum (Shanklin & Cahoon, 1998). Interestingly, our GWAS identified a *FAD2* gene (Csa01g013220) on chromosome 1 in camelina that is associated with the level of C18:1 desaturation. Three homeologous *FAD2* genes (Csa01g013220, Csa15g016000, and Csa19g016350) are present in the camelina genome (Hutcheon et al., 2010; Kang et al., 2011). Csa01g013220 corresponds to *CsFAD2‐2* that we characterized previously, which is a highly expressed homeolog in developing seed and when mutated significantly causes the reduction of the fatty acid desaturation (Kang et al., 2011). In addition, a previous gene dosage study on three copies of camelina *FAD2* genes revealed that mutations of *CsFAD2‐2* had higher impact of the oleic acid content than the other two homeologous alleles (Morineau et al., 2017). Our results indicate that *CsFAD2‐2* is the major determinant of the natural variation of polyunsaturated fatty acids in camelina seeds. The detection of this gene clearly demonstrates the efficiency and accuracy of the association mapping strategy used in this study.

### 4.3 The roles of the GDSL lipase in seed oil accumulation

In contrast to a simple genetic determinant of fatty acid composition, which has a well‐characterized biochemical path, the causal genes of the total seed OC and seed size are difficult to determine by the GWAS approach. This is expected because these latter traits are controlled by multiple genes involved in various developmental and metabolic processes (Chao et al., 2017; Li et al., 2019). Although a genome sequence of camelina is available (Kagale et al., 2014), which provided a reference for SNP mapping, the lack of a high‐quality reference genome of camelina may have also limited our ability to pinpoint candidate genes. Nevertheless, the identification of a candidate gene encoding a GDSL lipase suggests that it may be a key factor influencing OC. Homologs of GDSL lipases have been found to play important roles in seed oil accumulation in other brassicas such as *A. thaliana* (Chen et al., 2012) and canola (Ding et al., 2019; Karunarathna et al., 2020). The GDSL lipases constitute the largest lipase–esterase family in *A. thaliana* with more than 100 members, though only some of them have true fatty acyl hydrolytic activities (Lai et al., 2017; Wang et al., 2019). Disruption of the five GDSL genes called *SEED FATTY ACID REDUCER* (*SFAR1* through *5*) resulted in increased fatty acid content in *A. thaliana* seeds, while overexpression of each gene reduced the total seed fatty acid content (Chen et al., 2012). Overexpression of AtGDSL1 and BnGDSL1 promoted lipid catabolism and decreased the seed OC. Conversely, RNAi‐ mediated suppression of BnGDSL1 in canola increased the seed OC (Ding et al., 2019). The mechanisms of GDSL lipase in oil biosynthesis is not clear; however, it is known that lipid degradation occurs during seed maturation (Kelly et al., 2013). Reducing the rate of lipid turnover may thus increase the amount of oil being stored in seed.

### 4.4 An *FLC* may play a major role in flowering of spring camelina

Camelina is an annual crop with a monocarpic lifestyle. The DTF is an important factor for its adaptation to cropping systems. Most cultivated camelina do not require vernalization to flower, while some do and can be grown as a winter cover crop to provide oilseed production and ecosystem services such as reducing erosion, nutrient scavenging, and springtime weed suppression (Berti et al., 2016). The *FLC* gene is a central player in the vernalization process in *A. thaliana*, which blocks flowering by inhibiting genes required to switch the meristem from vegetative to floral development (Henderson & Dean, 2004). Three orthologous *FLC* genes are present in the camelina genome (Csa08g054450, Csa13g011890, and Csa20g015400). In a previous study, an *FLC* gene on chromosome 20 (Csa20g015400) was attributed to differentiate the spring and winter camelina (Anderson et al., 2018). To identify genes controlling flowering time in spring camelina, we used a RIL population that we previously developed by crossing two spring‐type accessions differing in a number of traits including flowering time (King et al., 2019). We detected major QTL controlling several important traits (Supplemental Table S5), most strikingly, the colocalization of a QTL on chromosome 8 with an *FLC* gene (Csa08g054450) (Figure 6b). Apparently, several genes are involved in the determination of flowering in camelina as suggested by other QTL regions across the genome. Although validation of these results is needed by further mapping studies and functional characterization of candidate genes, our study suggests that *CsFLC* on chromosome 8 may be the major player that governs the floral evocation in spring camelina.

## AUTHOR CONTRIBUTIONS

Huang Li: Formal analysis; Investigation; Methodology; Writing‐original draft; Writing‐review & editing. Xiao Hu: Methodology. Sujan Mamidi: Methodology. Cindy Chen: Methodology. Mojgan Amirebrahimi: Methodology. Indika Kahanda: Methodology; Supervision. Brendan Mumey: Methodology; Supervision. David Kudrna: Methodology. Jeremy Schmutz: Conceptualization; Project administration; Supervision. Jennifer Lachowiec: Writing‐review & editing. Chaofu Lu: Conceptualization; Funding acquisition; Project administration; Supervision; Writing‐review & editing

## CONFLICT OF INTEREST

The authors declare no conflict of interest.

## Supporting information



Supplemental Figure S1. Density of filtered SNPs in 222 *C. sativa* accessions across the genome.
Supplemental Figure S2. Population structure and genetic diversity estimate of 222 *C. sativa* accessions.
Supplemental Figure S3. Estimation of linkage disequilibrium (LD) decay rate in the C. sativa genome.
Supplemental Figure S4. Comparison of quantile–quantile (QQ) plots for different GWAS statistical methods.
Supplemental Figure S5. Correlation coefficients of seed oil content and fatty acid composition *C. sativa* accessions.
Supplemental Figure S6. Correlation coefficients of field and seed quality traits in the RIL population.
Supplemental Table S1. List of the 222 genome‐resequenced *Camelina sativa* accessions.
Supplemental Table S2. Descriptive statistics for phenotypic variation and heritability of field and seed quality traits in *C. sativa* accessions.
Supplemental Table S3. Summary of GWAS results for field and seed quality traits.
Supplemental Table S4. Descriptive statistics for phenotypic variation and heritability of field and seed quality traits in the RIL population.
Supplemental Table S5. Summary of QTL identified in the Suneson/Prytzh RIL population.
