# Supplementary material for: Genetic dissection of natural variation in oilseed traits of camelina by whole‐genome resequencing and QTL mapping
Source: Plant Genome. 2021 Jun 9;14(2):e20110. doi: 10.1002/tpg2.20110 (PMC12806864; doi:10.1002/tpg2.20110)
Supplement: Supplementary file 1 [file TPG2-14-e20110-s001.docx]

## Supporting Information

**Genetic Dissection of Natural Variation in Oilseed Traits of *Camelina sativa* by Whole-genome Resequencing and Quantitative Trait Loci Mapping**

Huang Li^1^, Xiao Hu^2^, John T. Lovell^3^, Paul P. Grabowski^3^, Sujan Mamidi^3^, Cindy Chen^4^, Mojgan Amirebrahimi^4^, Indika Kahanda^2^, Brendan Mumey^2^, Kerrie Barry^4^, David Kudrna^5^, Jeremy Schmutz^3,4^, Jennifer Lachowiec^1^, Chaofu Lu^1,*^

The following Supporting Information is available for this article:

**Fig. S1.** Density of filtered SNPs in 222 *C. sativa* accessions across the genome.

**Fig. S2.** Population structure and genetic diversity estimate of 222 *C. sativa* accessions.

**Fig. S3.** Estimation of linkage disequilibrium (LD) decay rate in the *C. sativa* genome.

**Fig. S4.** Comparison of quantile–quantile (QQ) plots for different GWAS statistical methods.

**Fig. S5.** Correlation coefficients of seed oil content and fatty acid composition *C. sativa* accessions.

**Fig. S6.** Correlation coefficients of field and seed quality traits in the RIL population.

**Table S1.** List of the 222 genome-resequenced *Camelina sativa* accessions.

**Table S2.** Descriptive statistics for phenotypic variation and heritability of field and seed quality traits in *C. sativa* accessions.

**Table S3.** Summary of GWAS results for field and seed quality traits.

**Table S4.** Descriptive statistics for phenotypic variation and heritability of field and seed quality traits in the RIL population.

**Table S5.** Summary of QTLs identified in the Suneson/Prytzh RIL population.

**Fig. S1.** Density of filtered SNPs in 222 *C. sativa* accessions across the genome.

(a)

(b)	(c)

Pairwise Fst values among sub-populations

| Group  (Sub-population) | 1 | 2 | 3 | Number of Lines |
| --- | --- | --- | --- | --- |
| 1 | — |  |  | 34 |
| 2 | 0.072 | — |  | 82 |
| 3 | 0.083 | 0.071 | — | 72 |
| 4 | 0.171 | 0.143 | 0.090 | 34 |

(d)

**Fig. S2.** Population structure and genetic diversity estimate of 222 *C. sativa* accessions. (a) Cross-Validation (CV) error in the population structure analysis for K**.** (b) Pairwise Fst values among 4 sub-populations (c) Priciple component analysis (PCA) showing clustered groups (d) fastSTRUCTURE analysis bar plots for K = 2 to K=12 showing population structure of different sub-populations.

**Fig. S3** Estimation of linkage disequilibrium (LD) decay rate in *C. sativa* genome

**Fig. S4.** Comparison of quantile-quantile (QQ) plots for different GWAS statistical methods

**Fig. S5.** Correlation coefficients of seed oil content and fatty acid composition in *C. sativa* accessions.

**Fig. S6.** Correlation coefficients of field and seed quality traits in the RIL population.

**Table S1**. List of the 222 genome-resequenced *C. sativa* accessions

| **Accession number** | **Accession name** | **Other name** | **Biological status** | **Country of origin** |
| --- | --- | --- | --- | --- |
| CS001 | Suneson | MT5 | Cultivar | United States |
| CS002 | Licalla |  | Advanced/improved cultivar | Germany |
| CS003 | Kirgizskij | CAM 7 | Advanced/improved cultivar | Kyrgyzstan |
| CS004 | Sortandinskij | CAM 8 | Advanced/improved cultivar | Former Soviet Union |
| CS007 | OmskijMestnyj | CAM 25 | Mutant | Former Soviet Union |
| CS008 | Czestochowska | CAM 28 | Advanced/improved cultivar | Poland |
| CS009 | Volynskaja1 | CAM 29 | Advanced/improved cultivar | Ukraine |
| CS010* | ZarjaSocializma2 | CAM 30 | Advanced/improved cultivar | Former Soviet Union |
| CS011* | CAM31 | CAM 31 | Traditional cultivar/landrace | Poland |
| CS013 | Bronowska1 | CAM 33 | Advanced/improved cultivar | Poland |
| CS015 | Ukrajinskij | CAM 35 | Advanced/improved cultivar | Former Soviet Union |
| CS016 | Wroclawska | CAM 36 | Advanced/improved cultivar | Poland |
| CS018 | CAM38 | CAM 38 | Traditional cultivar/landrace | Austria |
| CS019 | CAM39 | CAM 39 | Traditional cultivar/landrace | Austria |
| CS020 | Lindo | CAM 40 | Advanced/improved cultivar | Germany |
| CS022 | Ukrajinskaja | CAM 45 | Advanced/improved cultivar | Former Soviet Union |
| CS023 | CAM49 | CAM 49 |  | Poland |
| CS024 | CAM52 | CAM 52 | NA | Slovenia |
| CS025 | CAM55 | CAM 55 | NA | Austria |
| CS026 | OmskijMestnyi2 | CAM 56 | Mutant | Former Soviet Union |
| CS027 | Kirgizskij2 | CAM 57 | Advanced/improved cultivar | Former Soviet Union |
| CS028 | CAM58 | CAM 58 | Wild | Germany |
| CS030 | Boha1 | CAM 61 | Advanced/improved cultivar | Denmark |
| CS031 | CAME1 | CAM 63 | Advanced/improved cultivar | Swedenden |
| CS033 | CAM66 | CAM 66 | Advanced/improved cultivar | Germany |
| CS034 | Hoga1 | CAM 67 | Advanced/improved cultivar | Denmark |
| CS035 | Svalof | CAM 68 | Advanced/improved cultivar | Sweden |
| CS036 | CAM70 | CAM 70 | Wild | Germany |
| CS037 | Vniimk | CAM 72 | Advanced/improved cultivar | Former Soviet Union |
| CS038 | Kirkizska | CAM 73 | Advanced/improved cultivar | Former Soviet Union |
| CS039 | Czenstochwska1 | CAM 74 | Advanced/improved cultivar | Czech Republic |
| CS040* | Auslese2 | CAM 77 | Breeder's line | Former Soviet Union |
| CS041 | Auslese1 | CAM 78 | Breeder's line | Former Soviet Union |
| CS042 | PRFGL47 | CAM 79 | Breeding/research material | Germany |
| CS043 | PRFGL36 | CAM 80 | Breeding/research material | Germany |
| CS044 | PRFGL40 | CAM 81 | Breeding/research material | Germany |
| CS046 | PRFGL75 | CAM 83 | Breeding/research material | Germany |
| CS047 | PRFGL42 | CAM 84 | Breeding/research material | Germany |
| CS048 | PRFGL45 | CAM 85 | Breeding/research material | Germany |
| CS049 | PRFGL57 | CAM 86 | Breeding/research material | Germany |
| CS050 | PRFGL49 | CAM 87 | Breeding/research material | Germany |
| CS051 | PRFGL39 | CAM 88 | Breeding/research material | Germany |
| CS052 | PRFGL72 | CAM 89 | Breeding/research material | Germany |
| CS053 | PRFGL54 | CAM 90 | Breeding/research material | Germany |
| CS054 | PRFGL69 | CAM 91 | Breeding/research material | Germany |
| CS055 | PRFGL59 | CAM 92 | Breeding/research material | Germany |
| CS056 | PRFGL21 | CAM 95 | Breeding/research material | Germany |
| CS057 | PRFGL20 | CAM 96 | Breeding/research material | Germany |
| CS058 | PRFGL19 | CAM 98 | Breeding/research material | Germany |
| CS059 | PRFGL10 | CAM 99 | Breeding/research material | Germany |
| CS060 | PRFGL16 | CAM 101 | Breeding/research material | Germany |
| CS062 | PRFGL34 | CAM 104 | Breeding/research material | Germany |
| CS063 | Bronowska2 | CAM 108 | Breeding/research material | Poland |
| CS064 | Volynskaja2 | CAM 110 | Advanced/improved cultivar | Poland |
| CS065 | Omskaja | CAM 111 | Advanced/improved cultivar | Former Soviet Union |
| CS066 | PRFGL23 | CAM 112 | Breeding/research material | Germany |
| CS067 | PRFGL28 | CAM 114 | Breeding/research material | Germany |
| CS068 | CAM116 | CAM 116 | Wild | Belgium |
| CS069 | PRFGL27 | CAM 119 | Breeding/research material | Germany |
| CS070 | WroclawskaAMCSA70 | CAM 120 | Advanced/improved cultivar | Poland |
| CS071 | Czenstochwska2 | CAM 123 | Advanced/improved cultivar | Czech Republic |
| CS072 | PRFGL22 | CAM 124 | Breeding/research material | Germany |
| CS073 | PRFGL32 | CAM 131 | Breeding/research material | Germany |
| CS074 | CalenaAMCSA74 | CAM 134 | Advanced/improved cultivar | Germany |
| CS075 | CAM135 | CAM 135 | Wild | Germany |
| CS076 | Sortadinskij | CAM 136 | Advanced/improved cultivar | Poland |
| CS077 | CAM137 | CAM 137 | Wild | Denmark |
| CS078 | CAM138 | CAM 138 | Wild | NA |
| CS079 | CAM145 | CAM 145 | Wild | Germany |
| CS080 | STAMM13X14A | CAM 146 | Breeder's line | Germany |
| CS081 | STAMM09X13 | CAM 147 | Breeder's line | Germany |
| CS082 | CAM148 | CAM 148 | Wild | Bulgaria |
| CS083 | STAMM11X15 | CAM 149 | Breeder's line | Germany |
| CS084 | STAMM09X14 | CAM 150 | Breeder's line | Germany |
| CS085 | STAMM07X13B | CAM 151 | Breeder's line | Germany |
| CS086 | STAMM09X11 | CAM 152 | Breeder's line | Germany |
| CS087 | STAMM08X14 | CAM 153 | Breeder's line | Germany |
| CS088 | STAMM12X13 | CAM 154 | Breeder's line | Germany |
| CS089 | STAMM08X13 | CAM 155 | Breeder's line | Germany |
| CS090 | STAMM09X15 | CAM 156 | Breeder's line | Germany |
| CS091 | STAMM10X12 | CAM 157 | Breeder's line | Germany |
| CS092 | STAMM10X13 | CAM 158 | Breeder's line | Germany |
| CS093 | STAMM13X15 | CAM 159 | Breeder's line | Germany |
| CS094 | STAMM13X14B | CAM 160 | Breeder's line | Germany |
| CS095 | STAMM10X15 | CAM 161 | Breeder's line | Germany |
| CS096 | STAMM06X13B | CAM 162 | Breeder's line | Germany |
| CS098 | STAMM06X10A | CAM 164 | Breeder's line | Germany |
| CS099 | STAMM04X05 | CAM 165 | Breeder's line | Germany |
| CS101 | STAMM06X14C | CAM 167 | Breeder's line | Germany |
| CS102 | CAM168 | CAM 168 | Wild | NA |
| CS103 | Vnttmk17 | CAM 169 | Breeding/research material | Former Soviet Union |
| CS104 | CAM170 | CAM 170 | Wild | Poland |
| CS105 | Guillemeteau | CAM 171 | Advanced/improved cultivar | NA |
| CS106* | Zarjasocialisma1 | CAM 172 | Advanced/improved cultivar | Former Soviet Union |
| CS107 | Irkutskajamestnaja | CAM 173 | Traditional cultivar/landrace | Former Soviet Union |
| CS108 | Voronezskij3491 | CAM 174 | Advanced/improved cultivar | NA |
| CS109 | CAM175 | CAM 175 | Wild | Sweden |
| CS110 | Bavaria | CAM 180 | advanced/improved cultivar | Germany |
| CS111 | CAM182 | CAM 182 | Breeding/research material | NA |
| CS112 | CAM187 | CAM 187 | Wild | Spain |
| CS114 | STAMM06X13A | CAM 192 | Breeder's line | Germany |
| CS115 | STAMM06X15B | CAM 193 | Breeder's line | Germany |
| CS116 | STAMM06X15A | CAM 194 | Breeder's line | Germany |
| CS117 | STAMM06X08C | CAM 195 | Breeder's line | Germany |
| CS118 | STAMM06X10B | CAM 196 | Breeder's line | Germany |
| CS119 | STAMM02X12A | CAM 197 | Breeder's line | Germany |
| CS120 | STAMM06X07B | CAM 198 | Breeder's line | Germany |
| CS121 | STAMM07X11A | CAM 199 | Breeder's line | Germany |
| CS122 | STAMM06X08A | CAM 200 | Breeder's line | Germany |
| CS123 | STAMM03X13 | CAM 201 | Breeder's line | Germany |
| CS125 | STAMM02X11C | CAM 203 | Breeder's line | Germany |
| CS126 | STAMM01X13 | CAM 204 | Breeder's line | Germany |
| CS127 | STAMM06X09 | CAM 205 | Breeder's line | Germany |
| CS128 | STAMM05X11 | CAM 206 | Breeder's line | Germany |
| CS129 | STAMM02X09 | CAM 207 | Breeder's line | Germany |
| CS131 | STAMM01X08 | CAM 209 | Breeder's line | Germany |
| CS132 | STAMM14X15 | CAM 210 | Breeder's line | Germany |
| CS133 | STAMM06X12B | CAM 211 | Breeder's line | Germany |
| CS134 | STAMM02X11A | CAM 212 | Breeder's line | Germany |
| CS136 | STAMM04X13 | CAM 214 | Breeder's line | Germany |
| CS137 | CAM216 | CAM 216 | Traditional cultivar/landrace | Former Soviet Union |
| CS138 | CAM218 | CAM 218 | Traditional cultivar/landrace | Former Soviet Union |
| CS139 | CAM219 | CAM 219 | Traditional cultivar/landrace | Former Soviet Union |
| CS140 | CAME2 | CAM 220 | Traditional cultivar/landrace | Sweden |
| CS141 | Voronezskij3392 | CAM 221 | Advanced/improved cultivar | Former Soviet Union |
| CS142 | Borow1 | CAM 223 | Advanced/improved cultivar | Poland |
| CS143 | Krajova | CAM 225 | Advanced/improved cultivar | NA |
| CS144 | Vnjjmk17 | CAM 226 | Breeding/research material | Former Soviet Union |
| CS145 | PRFGL81 | CAM 228 | Breeding/research material | Germany |
| CS146 | PRFGL96 | CAM 229 | Breeding/research material | Poland |
| CS147 | PRFGL88 | CAM 230 | Breeding/research material | Germany |
| CS148 | PRFGL77 | CAM 231 | Breeding/research material | Germany |
| CS149 | PRFGL85 | CAM 232 | Breeding/research material | Germany |
| CS150 | PRFGL95 | CAM 233 | Breeding/research material | Germany |
| CS151 | PRFGL76 | CAM 234 | Breeding/research material | Germany |
| CS152 | PRFGL87 | CAM 235 | Breeding/research material | Germany |
| CS153 | PRFGL91 | CAM 236 | Breeding/research material | NA |
| CS154 | PRFGL82 | CAM 237 | Breeding/research material | Germany |
| CS155 | PRFGL80 | CAM 239 | Breeding/research material | Germany |
| CS156 | PRFGL94 | CAM 240 | Breeding/research material | Germany |
| CS157 | PRFGL78 | CAM 241 | Breeding/research material | Germany |
| CS158 | PRFGL92 | CAM 242 | Breeding/research material | Germany |
| CS159 | CAM243 | CAM 243 |  | Germany |
| CS160 | STAMM02X12B | CAM 244 | Wild | Former Soviet Union |
| CS162 | STAMM06X08B | CAM 246 | Breeder's line | Germany |
| CS163 | STAMM07X13A | CAM 247 | Breeder's line | Germany |
| CS164 | STAMM06X14A | CAM 248 | Breeder's line | Germany |
| CS165 | STAMM03X07 | CAM 250 | Breeder's line | Germany |
| CS166 | STAMM05X14A | CAM 251 | Breeder's line | Germany |
| CS167 | STAMM02X10B | CAM 252 | Breeder's line | Germany |
| CS168 | STAMM06X14B | CAM 253 | Breeder's line | Germany |
| CS170 | STAMM02X08B | CAM 255 | Breeder's line | Germany |
| CS171 | STAMM05X14C | CAM 256 | Breeder's line | Germany |
| CS172 | STAMM06X11B | CAM 257 | Breeder's line | Germany |
| CS173 | STAMM06X13C | CAM 258 | Breeder's line | Germany |
| CS174 | CAM259 | CAM 259 | Wild | Bulgaria |
| CS175 | CAM260 | CAM 260 | Wild | Bulgaria |
| CS176 | CAM261 | CAM 261 | Wild | Bulgaria |
| CS177 | CAM262 | CAM 262 | Wild | Romania |
| CS178 | CAM264 | CAM 264 | Wild | Bulgaria |
| CS179 | CAM266 | CAM 266 | Wild | Former Soviet Union |
| CS180 | CAM268 | CAM 268 | Wild | Bulgaria |
| CS181 | CAM269 | CAM 269 | Wild | Great Britain |
| CS182 | CAM270 | CAM 270 | Wild | Czech Republic |
| CS183 | STAMM05X14D | CAM 271 | Breeder's line | Germany |
| CS184 | CAM272 | CAM 272 | Wild | Belgium |
| CS185 | CAM273 | CAM 273 | Wild | Sweden |
| CS188 | CAM276 | CAM 276 | Wild | Bulgaria |
| CS189 | Calinka | CAM 277 | NA | Germany |
| CS190 | STAMM02X11B | CAM 278 | Breeder's line | Germany |
| CS191 | GE201101 | Ames 31231 | Wild | Georgia |
| CS192 | GE201105 | Ames 31232 | Wild | Georgia |
| CS193 | Vniimk17 | PI 258366 | Early variety | Former Soviet Union |
| CS194 | Voronezh349 | PI 258367 | Early variety | Former Soviet Union |
| CS195 | No401 | PI 304268 | Breeding line | Sweden |
| CS196 | No402 | PI 304269 | Breeding line | Sweden |
| CS199 | Borow2 | PI 311735 | donated/collected seed | Poland |
| CS200* | Przybrodzka | PI 311736 | donated/collected seed | Poland |
| CS201 | 163207372 | PI 597833 | donated/collected seed | Denmark |
| CS202 | CR47665 | PI 633192 | donated/collected seed | Germany |
| CS203 | CR49294a | PI 633193 | donated/collected seed | Germany |
| CS204 | GiessenNr3 | PI 633194 | donated/collected seed | Germany |
| CS206 | CAME3 | PI 650140 | donated/collected seed | Germany |
| CS207 | NU52279 | PI 650141 | donated/collected seed | United States |
| CS208 | CS163 | PI 650142 | donated/collected seed | Denmark |
| CS209* | CSCroo | PI 650143 | donated/collected seed | Germany |
| CS210 | Boha2 | PI 650144 | donated/collected seed | Denmark |
| CS211 | BRSCHW28347 | PI 650145 | donated/collected seed | Germany |
| CS212 | BRSCHW30021 | PI 650146 | donated/collected seed | Sweden |
| CS213 | CAME4 | PI 650147 | donated/collected seed | Sweden |
| CS214 | Giessen3 | PI 650148 | donated/collected seed | Germany |
| CS215 | Giessen4 | PI 650149 | donated/collected seed | Germany |
| CS216 | Hoga2 | PI 650150 | donated/collected seed | Denmark |
| CS217 | SvalofAMCSA217 | PI 650151 | donated/collected seed | Sweden |
| CS218 | CPSCAM23 | PI 650152 | donated/collected seed | Germany |
| CS219 | CPSCAM10 | PI 650153 | donated/collected seed | Former Soviet Union |
| CS220 | CSSCAM25 | PI 650154 | donated/collected seed | Former Soviet Union |
| CS221* | CSSCAM27 | PI 650155 | donated/collected seed | Poland |
| CS222 | CSSCAM29 | PI 650156 | donated/collected seed | Former Soviet Union |
| CS223 | CSSCAM30 | PI 650157 | donated/collected seed | Former Soviet Union |
| CS224* | CSSCAM31 | PI 650158 | donated/collected seed | Poland |
| CS225 | CSSCAM33 | PI 650159 | donated/collected seed | Poland |
| CS226 | CSSCAM34 | PI 650160 | donated/collected seed | Former Soviet Union |
| CS227 | CSSCAM35 | PI 650161 | donated/collected seed | Former Soviet Union |
| CS228 | CSSCAM36 | PI 650162 | donated/collected seed | Poland |
| CS229 | CSSCAM37 | PI 650163 | donated/collected seed | Former Soviet Union |
| CS230 | CSSCAM38 | PI 650164 | donated/collected seed | Austria |
| CS231 | CSSCAM7 | PI 650165 | donated/collected seed | Former Soviet Union |
| CS232 | CSSCAM8 | PI 650166 | donated/collected seed | Former Soviet Union |
| CS233 | Seminum144 | PI 650167 | donated/collected seed | Poland |
| CS234* | NE20061 | PI 650168 | Advanced/improved cultivar | United States |
| CS235 | AMES29309 | PI 652885 | donated/collected seed | Slovenia |
| CS236 | AMES29310 | PI 652886 | donated/collected seed | Slovenia |
| CS245 | MT1 |  | Breeder's line | United States |
| CS246 | Blaine Creek | MT3 | Breeder's line | United States |
| CS248 | Celine |  | Advanced/improved cultivar | France |
| CS249 | Cheyenne |  | Advanced/improved cultivar | United States |
| CS250 | Ligena |  | Advanced/improved cultivar | Germany |
| CS251 | Prytzh | MT102 | Big seed variety | Ukraine |
| CS252 | MT148 |  | Breeder's line | United States |
| CS253 | MT229 |  | Breeder's line | United States |
| CS254 | MT402 |  | Breeder's line | United States |

***** Indicates accessions that didn’t bolt in both years of field trial. They are potential winter types that requires vernalization to flower.

| Trait | (Mean ± SD) | Range | Year-to-year  Correlation | | Heritability  (H²) | | | | | | | | |  |  |  |  |  |  |
| --- | --- | --- | --- | --- | --- | --- | --- | --- | --- | --- | --- | --- | --- | --- | --- | --- | --- | --- | --- |
| SAS(mm²) | 1.33±0.16 | 0.99-1.96 | | 0.84** | | | 0.94 | | | | |  | | | | | |  |  |
| SL(mm) | 1.76±0.11 | 1.54-2.24 | | 0.86** | | | 0.95 | | |  | | | | | | | |  |  |
| SW(mm) | 1.00±0.05 | 0.87-1.13 | | 0.81** | | | 0.93 | |  | | | | | |  |  |  |  |  |
| TSW(g) | 1.17±0.16 | 0.87-1.74 | | 0.86** | | | 0.93 | | | |  | | | | | |  |  |  |
| PAS (mm²) | 21.97±2.05 | 17.94-33.20 | | 0.62** | | | 0.89 | |  | | | | | |  |  |  |  |  |
| PL (mm) | 7.55±0.45 | 6.47-8.86 | | 0.66** | | | 0.88 | |  | | | | | |  |  |  |  |  |
| PW (mm) | 4.23±0.23 | 3.73-5.41 | | 0.66** | | | 0.89 | | | | | |  | | |  |  |  |  |
| SPP | 13.52±1.04 | 9.95-16.40 | | 0.41** | | | 0.73 | | | | | |  | | |  |  |  |  |
| OC (%) | 38.17±0.73 | 35.66-40.28 | | 0.43** | | 0.38 | |  | | | | | | | | | | |  |
| DTF (days) | 52.27±1.59 | 46.79-57.49 | | 0.35** | | 0.72 | |  | | | | | | | | | | |  |
| DTM (days) | 97.08±1.95 | 90.95-102.82 | | 0.44** | | 0.66 | |  | | | | | | | | | | |  |
| PH (cm) | 76.19±3.78 | 55.20-83.64 | | 0.39** | | 0.22 | | | | | | | | | | |  |  |  |

**Table S2.** Descriptive statistics for phenotypic variation and heritability of field and seed quality traits in *C. sativa* accessions.

**Table S3.** Summary of GWAS results for field and seed quality traits.

| **Trait** | **Loci** | **SNP location** | **P value** | | **MAF** | | **Effect** | | |
| --- | --- | --- | --- | --- | --- | --- | --- | --- | --- |
| SAS | 1 | **Chr7_15494586** | | 3.77E-17 | | 0.134 | | 0.037 |  |
|  | 2 | Chr1_4450342 | | 2.99E-12 | | 0.100 | | -0.041 |  |
|  | 3 | **Chr9_33876784** | | 2.98E-09 | | 0.201 | | 0.024 |  |
|  | 4 | **Chr12_8071010** | | 3.26E-08 | | 0.118 | | -0.023 |  |
|  | 5 | Chr20_10493759 | | 8.72E-08 | | 0.361 | | -0.017 |  |
|  | 6 | Chr2_701071 | | 1.08E-07 | | 0.252 | | 0.044 |  |
|  | 7 | **Chr20_4705948** | | 1.43E-07 | | 0.188 | | 0.038 |  |
| SL | 1 | **Chr12_8071010** | | 3.07E-16 | | 0.185 | | -0.031 |  |
|  | 2 | **Chr7_15494586** | | 1.41E-09 | | 0.327 | | 0.017 |  |
|  | 3 | Chr18_12533726 | | 6.89E-08 | | 0.103 | | -0.022 |  |
|  | 4 | Chr8_24935587 | | 2.15E-07 | | 0.217 | | 0.023 |  |
| SW | 1 | Chr3_9124793 | | 9.90E-10 | | 0.107 | | -0.014 |  |
|  | 2 | Chr19_8611572 | | 1.69E-08 | | 0.336 | | 0.011 |  |
|  | 3 | Chr9_33876784 | | 1.87E-08 | | 0.432 | | 0.008 |  |
|  | 4 | **Chr20_4705948** | | 2.90E-08 | | 0.121 | | 0.016 |  |
|  | 5 | Chr1_2581425 | | 4.34E-08 | | 0.103 | | -0.011 |  |
|  | 6 | Chr9_32630657 | | 1.79E-07 | | 0.456 | | -0.008 |  |
| PAS | 1 | **Chr7_24295852** | | 1.24E-12 | | 0.112 | | -1.001 |  |
|  | 2 | Chr19_3025785 | | 9.84E-09 | | 0.474 | | 0.500 |  |
|  | 3 | Chr5_14536116 | | 2.15E-07 | | 0.173 | | -0.484 |  |
| PL | 1 | Chr7_14318121 | | 6.37E-11 | | 0.217 | | 0.135 |  |
|  | 2 | Chr12_4754022 | | 4.05E-10 | | 0.481 | | 0.122 |  |
|  | 3 | Chr13_16869038 | | 7.30E-10 | | 0.222 | | -0.192 |  |
|  | 4 | Chr5_16896860 | | 3.01E-09 | | 0.147 | | 0.187 |  |
|  | 5 | Chr6_13707922 | | 1.13E-08 | | 0.451 | | 0.109 |  |
|  | 6 | Chr16_26230101 | | 2.10E-08 | | 0.112 | | 0.189 |  |
|  | 7 | Chr9_7139766 | | 1.00E-07 | | 0.157 | | -0.160 |  |
|  | 8 | Chr8_10452428 | | 1.37E-07 | | 0.126 | | 0.177 |  |
| PW | 1 | **Chr7_24297173** | | 1.85E-08 | | 0.322 | | 0.061 |  |
|  | 2 | Chr13_7864480 | | 3.51E-08 | | 0.161 | | -0.078 |  |
|  | 3 | Chr11_30590431 | | 1.14E-07 | | 0.107 | | 0.103 |  |
|  | 4 | Chr11_13929956 | | 1.16E-07 | | 0.245 | | -0.092 |  |
|  | 5 | Chr3_10262729 | | 1.95E-07 | | 0.414 | | -0.049 |  |
| TSW | 1 | Chr20_26075941 | | 1.79E-14 | | 0.138 | | -0.046 |  |
|  | 2 | Chr15_7077493 | | 1.83E-11 | | 0.488 | | 0.029 |  |
|  | 3 | Chr11_33828272 | | 4.00E-10 | | 0.143 | | 0.053 |  |
|  | 4 | Chr9_25325192 | | 3.04E-09 | | 0.143 | | -0.041 |  |
|  | 5 | Chr15_20492121 | | 1.22E-08 | | 0.114 | | 0.044 |  |
|  | 6 | Chr16_5799612 | | 1.27E-07 | | 0.182 | | 0.036 |  |
|  | 7 | Chr18_1674742 | | 1.60E-07 | | 0.131 | | -0.041 |  |
| SPP | 1 | Chr17_27673565 | | 7.31E-13 | | 0.284 | | -0.422 |  |
|  | 2 | Chr1_7959098 | | 4.03E-12 | | 0.200 | | 0.341 |  |
|  | 3 | Chr19_21487643 | | 1.09E-09 | | 0.453 | | 0.256 |  |
|  | 4 | Chr11_5333523 | | 2.37E-08 | | 0.268 | | -0.215 |  |
|  | 5 | Chr20_10488340 | | 6.30E-08 | | 0.324 | | -0.188 |  |
|  | 6 | Chr11_9989376 | | 8.62E-08 | | 0.153 | | -0.485 |  |
|  | 7 | Chr7_12582732 | | 1.52E-07 | | 0.488 | | 0.227 |  |
| OC | 1 | Chr16_17714861 | | 2.15E-11 | | 0.446 | | 0.192 |  |
|  | 2 | Chr6_6200255 | | 3.51E-10 | | 0.207 | | -0.327 |  |
|  | 3 | Chr2_27306963 | | 1.89E-08 | | 0.481 | | 0.190 |  |
| C18:1 | 1 | Chr1_5202923 | | 1.05E-27 | | 0.134 | | -0.741 |  |
|  | 2 | Chr20_14610351 | | 1.96E-12 | | 0.100 | | -0.389 |  |
|  | 3 | Chr14_18836492 | | 9.60E-11 | | 0.201 | | 0.352 |  |
|  | 4 | Chr1_11441965 | | 1.26E-08 | | 0.118 | | -0.344 |  |
|  | 5 | Chr18_18280373 | | 1.53E-08 | | 0.361 | | -0.184 |  |
|  | 6 | Chr9_2842939 | | 5.02E-08 | | 0.252 | | 0.174 |  |
|  | 7 | Chr8_16871897 | | 1.56E-07 | | 0.188 | | -0.267 |  |
| C18:2 | 1 | Chr1_5202923 | | 1.45E-17 | | 0.134 | | 0.636 |  |
|  | 2 | Chr9_23819599 | | 4.00E-08 | | 0.134 | | 0.217 |  |
|  | 3 | Chr8_27710476 | | 8.43E-08 | | 0.204 | | 0.297 |  |
| PH | 1 | Chr20_5404348 | | 2.15E-10 | | 0.151 | | 1.700 |  |
|  | 2 | Chr8_26696501 | | 5.20E-10 | | 0.124 | | -1.926 |  |
|  | 3 | Chr16_21397328 | | 1.03E-08 | | 0.188 | | -1.575 |  |

| Trait | Pryzeth  (Mean ± SD) | Suneson  (Mean ± SD) | RIL  (Mean ± SD) | | Range | Heritability  (H²) | | | | |
| --- | --- | --- | --- | --- | --- | --- | --- | --- | --- | --- |
| DTF (day) | 43.54±1.54 | 48.24±0.92 | 45.08±2.26 | | 39.82-52.51 | 0.89 | |  |  |  |
| DTM (day) | 87.40±1.52 | 93.65±1.92 | 89.20±1.63 | | 85.64-94.09 | 0.67 |  |  |  |  |
| PH (cm) | 73.54±4.25 | 79.96±6.45 | 72.39±3.17 | | 63.80-79.42 | 0.68 |  |  |  |  |
| SAS (mm²) | 2.23±0.12 | 1.35±0.04 | 1.60±0.10 | | 1.39-1.97 | 0.80 | | |  |  |
| SL (mm) | 2.40±0.06 | 1.75±0.03 | 1.97±0.07 | | 1.79-2.17 | 0.87 |  |  |  |  |
| SW (mm) | 1.17±0.04 | 1.00±0.03 | 1.03±0.03 | | 0.97-1.14 | 0.85 | |  |  |  |
| TSW (g) | 1.84±0.22 | 1.07±0.07 | 1.32±0.08 | | 1.14-1.60 | 0.66 | | | |  |
| PAS (mm²) | 37.16±2.10 | 20.74±1.57 | 26.63±2.22 | | 20.48-33.84 | 0.89 | |  |  |  |
| PL (mm) | 9.95±0.62 | 7.98±0.51 | 8.30±0.52 | | 6.43-9.80 | 0.89 | |  |  |  |
| PW (mm) | 5.66±0.21 | 3.94±0.15 | 4.70±0.20 | | 4.16-5.27 | 0.87 | | | |  |
| SPP | 13.19±1.14 | 11.70±1.53 | 14.15±1.32 | | 10.43-17.99 | 0.84 | | | |  |
| OC (%) | 35.34±1.16 | 37.99±2.10 | | 38.01± 0.89 | 34.64-40.34 | 0.64 | | | |  |

**Table S4.** Descriptive statistics for phenotypic variation and heritability of field and seed quality traits in the RIL population.

**Table S5**. Summary of QTLs identified in the Suneson/Prytzh RIL population

| Trait | QTL | Marker interval  (location) | | Physical  interval  (kb) | Genetic  interval  (cM) | LOD | PVE (%) | Add.  effect | Overlapping  traits | | |
| --- | --- | --- | --- | --- | --- | --- | --- | --- | --- | --- | --- |
| DTF | DTF7.1 | Chr7_23573660-23788933 | | 215 | 2.3 | 5.6 | 9.5 | -0.71 | DTM; PH | |  |
|  | DTF8.1 | | Chr8_23184133-23526054 | 342 | 3.3 | 12.4 | 20 | 1.03 | DTM | |  |
|  | DTF17.1 | | Chr17_10514009-10776469 | 262 | 1.7 | 7.3 | 12.2 | 0.80 | DTM; SAS; SL;  PL;TSW | |  |
| PH | PH7.1 | | Chr7_23573660-23788933 | 215 | 2.3 | 5.9 | 10 | -1.01 | DTF; DTM |  |  |
|  | PH8.1 | | Chr8_23064384-23184133 | 120 | 1.8 | 5.9 | 9.3 | 0.99 |  |  |  |
|  | PH10.1 | | Chr10_11425494-11682907 | 258 | 2.3 | 10.9 | 17.7 | -1.36 |  |  |  |
|  | PH17.1 | | Chr17_10584752-10878217 | 294 | 1.5 | 5.9 | 9.3 | 0.98 |  |  |  |
|  | PH20.1 | | Chr20_2670140-2970692 | 300 | 1.8 | 4.0 | 6.9 | 0.83 |  |  |  |
| SAS | **SAS1.1** | | Chr1_4151406- 4330807 | 179 | 1.2 | 4.3 | 7.4 | -0.03 | SL; TSW |  |  |
|  | SAS2.1 | | Chr2_22073621- 22279417 | 209 | 2.1 | 4.6 | 8.0 | -0.03 | TSW |  |  |
|  | SAS6.1 | | Chr6_19349377- 19567197 | 218 | 1.6 | 4.5 | 7.7 | -0.03 | SL |  |  |
|  | **SAS16.1** | | Chr16_3436751- 3443420 | 67 | 2.5 | 5.3 | 9.1 | -0.03 | SL; SW; TSW |  |  |
|  | **SAS17.1** | | Chr17_10514009- 10776469 | 262 | 1.7 | 7.9 | 13.2 | -0.04 | DTF; DTM; SL; PL; TSW |  |  |
|  | SAS18.1 | | Chr18_15839151- 15895092 | 56 | 2.0 | 4.3 | 7.4 | -0.03 | SL; PAS; PW; TSW |  |  |
| PAS | PAS1.1 | | Chr1_7385829- 7613515 | 228 | 1.8 | 4.0 | 7.0 | -0.59 |  |  |  |
|  | **PAS5.1** | | Chr5_27151489- 29018975 | 1867 | 1.6 | 4.9 | 8.4 | -0.65 |  |  |  |
|  | PAS11.1 | | Chr11_34309087- 35093956 | 785 | 2.0 | 4.5 | 7.7 | -0.63 |  |  |  |
|  | **PAS18.1** | | Chr18_15723745- 15839151 | 115 | 1.8 | 5.9 | 10.0 | -0.72 | SAS; SL; PW; TSW |  |  |
|  | PAS20.1 | | Chr20_4273360- 4423773 | 150 | 2.1 | 5.4 | 9.2 | -0.69 | PL; PW; SL |  |  |
| OC | **TOC2.1** | | Chr2_25095743- 25212357 | 117 | 1.8 | 5.1 | 13.0 | 0.33 |  |  |  |
|  | **TOC9.1** | | Chr9_25774537- 26093584 | 319 | 1.5 | 7.5 | 18.3 | -0.41 |  |  |  |

QTLs also identified in previous study (King et al. 2019) are shown in bold. LOD, logarithm of odds; PVE, phenotypic variation explained.
